# Does practice make perfect? Laparoscopic training mainly improves motion efficiency: a prospective trial

**DOI:** 10.1007/s13304-023-01511-w

**Published:** 2023-05-09

**Authors:** Felix von Bechtolsheim, Stefanie Petzsch, Sofia Schmidt, Alfred Schneider, Sebastian Bodenstedt, Isabel Funke, Stefanie Speidel, Olga Radulova-Mauersberger, Marius Distler, Jürgen Weitz, Soeren Torge Mees, Florian Oehme

**Affiliations:** 1 Department for Visceral, Thoracic and Vascular Surgery, Faculty of Medicine and University Hospital Carl Gustav Carus, Technische Universität Dresden, Dresden, Germany; 2 Centre for Tactile Internet with Human-in-the-Loop (CeTI), Technische Universität Dresden, Dresden, Germany; 3 Division of Translational Surgical Oncology, National Center for Tumor Diseases (NCT) Dresden, Technische Universität Dresden, Dresden, Germany; 4 Department of General and Visceral Surgery, Städtisches Klinikum, Friedrichstraße 41, 01067 Dresden, Germany

**Keywords:** Learning curve, Laparoscopic skill analysis, Laparoscopic motion analysis, Proficiency-based learning, Individual hands

## Abstract

Training improves skills in minimally invasive surgery. This study aimed to investigate the learning curves of complex motion parameters for both hands during a standardized training course using a novel measurement tool. An additional focus was placed on the parameters representing surgical safety and precision. Fifty-six laparoscopic novices participated in a training course on the basic skills of minimally invasive surgery based on a modified Fundamentals of Laparoscopic Surgery (FLS) curriculum. Before, twice during, and once after the practical lessons, all participants had to perform four laparoscopic tasks (peg transfer, precision cut, balloon resection, and laparoscopic suture and knot), which were recorded and analyzed using an instrument motion analysis system. Participants significantly improved the time per task for all four tasks (all *p* < 0.001). The individual instrument path length decreased significantly for the dominant and non-dominant hands in all four tasks. Similarly, both hands became significantly faster in all tasks, with the exception of the non-dominant hand in the precision cut task. In terms of relative idle time, only in the peg transfer task did both hands improve significantly, while in the precision cut task, only the dominant hand performed better. In contrast, the motion volume of both hands combined was reduced in only one task (precision cut, *p* = 0.01), whereas no significant improvement in the relative time of instruments being out of view was observed. FLS-based skills training increases motion efficiency primarily by increasing speed and reducing idle time and path length. Parameters relevant for surgical safety and precision (motion volume and relative time of instruments being out of view) are minimally affected by short-term training. Consequently, surgical training should also focus on safety and precision-related parameters, and assessment of these parameters should be incorporated into basic skill training accordingly.

**Supplementary Information:**

The online version contains supplementary material available at 10.1007/s13304-023-01511-w.

## Introduction

The foundation for surgical training dates back to Theodor Billroth, who trained his students until he certified them to have sufficient competence to perform surgical activities independently [1]. William Halstedt adopted this concept after a study tour through Europe and established the first American residency program [2]. Nowadays, a structured and mandatory surgical training program is implemented in most countries [3].

The underlying assumption is that no one is born as a perfect surgeon. Therefore, education and training in surgical skills and performance are necessary. Darzi and Mackay consider the technical performance of a surgeon to be a combination of three aspects: knowledge, judgement, and dexterity [4]. While knowledge and judgement depend largely on theoretical education and experience and can therefore only be trained to a limited extent, dexterity can be actively improved through training.

The need for surgical training is particularly evident in minimally invasive procedures, as the technical circumstances present a challenge to surgeons [5]. The view through an endoscope significantly limits depth perception and overview [6]. In addition, the fulcrum effect (i.e. the inverted movement of the instruments) and lack of haptics complicate the interactions between surgeon, instruments, and tissue [7, 8]. Therefore, it is not surprising that skills acquired through open surgical procedures are difficult to transfer to minimally invasive procedures [9].

Thus, training is highly recommended or even mandatory for surgeons who wish to perform minimally invasive procedures. For ethical, safety, and efficiency reasons, structural training on real procedures in the operating room is often avoided [3]. Subsequently, many curricula for education and simulation-based training of minimally invasive surgery have been developed [3, 10, 11]. Such training can positively impact the learning curve during real laparoscopic procedures [12].

For many training curricula, the time per task is one of the key metrics to evaluate the surgical performance of trainees [13]. A better procedural time might correlate with surgical experience, but it is still considered a crude and indirect measure of technical skill [14]. With the introduction of virtual reality simulators, it became possible to measure much more complex variables, such as path length, number of movements, or speed [13]. This accumulation of different variables allows a much more deeper and differentiated insight into the learning curves of laparoscopic skills. Some publications have shown that the measurement of selected motion variables is also possible in (non-virtual) box trainers [15, 16]. This provides objective measurements in a more realistic scenario regarding haptics, depth perception, and interactions between instruments and tasks.

Nevertheless, knowledge regarding the dexterity learning curve, the very essence of surgical skill development, is mostly vague. This study aimed to evaluate the learning curve of selected motion parameters during a standardized training course in minimally invasive surgery for medical students in a non-virtual setting. There was a distinctive focus on differences between the dominant and non-dominant hands as well as on safety-related behavior, such as not visualizing both instruments.

## Materials and methods

All procedures performed in this trial were in accordance with the the 1964 Helsinki declaration and its later amendments. The experimental protocol was approved by the local ethics committee of TU Dresden (decision number EK 416092015). All participants provided informed consent prior to their participation. This article was written in accordance with the CONSORT statement [17].

### Participants

A total of 56 medical students were included in this trial after providing consent for participation and after appropriate information was provided by the principal investigators.

All students participated in an elective course for training in minimally invasive surgery. The training followed a standardized curriculum based on a modified FLS curriculum. Training was conducted until all students reached proficiency. This proficiency level was based on the average task completion time of three surgical residents. In detail, these proficiency thresholds were a completion time below 120 s for Peg transfer, 240 s for balloon resection and 300 s for both, precision cutting and laparoscopic suture and knot task. Participants had to reach the proficiency threshold twice during consecutive attempts. Both training and proficiency levels were described in detail in previous publications [18].

A questionnaire asking for basic participant information had to be completed at the beginning of the course by every participant.

### Training course

**Fig. 1:**
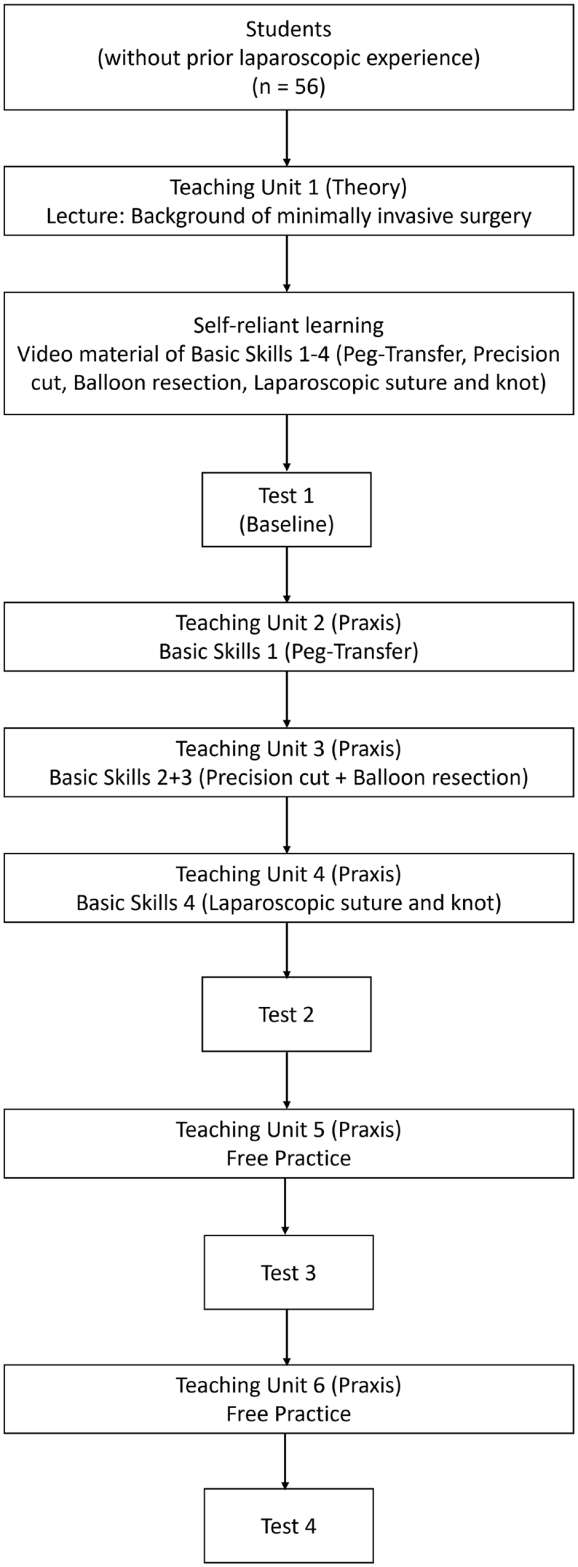
Schematic curriculum of the training course

A training course consisted of a maximum of 24 participants. A total of three training courses were included in the trial presented here. Each training course was conducted over a total of six sessions (Fig. 1):

1. Theoretical introduction to laparoscopic techniques and instruments (one session). After the theoretical introduction, the students were given access to the teaching videos for each task. These videos demonstrated the perfect execution of each task. The most common mistakes and potential pitfalls were also displayed, along with instructions to avoid such mistakes.
2. Practical introduction and hands-on training of all four tasks separately (three sessions). In the first training session, only peg transfer was demonstrated and performed. Subsequently, in the second training session, precision cut and balloon resection were performed. Eventually, the laparoscopic suture and knot task were performed during another separate session. In each session, the respective tasks were explained and demonstrated by at least two experienced surgeons.
3. Free training (two sessions). In the last two sessions, students had the opportunity to practice on the tasks independently and at their own discretion to improve their performance individually until reaching the respective level of proficiency for each task.

At any given moment in the course, at least two experienced and specifically trained surgeons were present and provided direct feedback for the respective task. A particular focus was placed on safety-relevant behaviors, such as avoidance of out-of-view movement or wide and unnecessary instrument movements.

### Scheduled tests

All participants were tested four times during laparoscopic training. The first test took place after the theoretical introduction and video teaching. Therefore, this test represents a baseline for laparoscopic performance before students were trained in minimally invasive surgery. The second test took place after Session 4; at which point all students had been introduced to all four tasks. After Session 5, the first free training session, the third test was conducted, and eventually, the fourth test was scheduled after the last training session.

### Instrument motion analysis (IMA)

Instrument motion analysis (IMA) consisted of a box trainer (Laparo Aspire®) and an optical tracking system (NDI Polaris®) with two infrared cameras, which tracked the motion of marker spheres attached to standard laparoscopic instruments (forceps, Overholt, scissor and needle holder by Fa. Storz). The differentiation of instruments and their respective sides (non-dominant/dominant hand) was enabled by using different patterns of marker spheres on the various instruments and by analyzing the initial questionnaire. The instrument tips were the point of reference for the calculated motion in the three-dimensional space. Their movements were calculated based on the tracked motions of the marker spheres at the handles of the instruments. The collected data enabled the calculation of motion volume, percentage of task time the instruments were out of endoscopic view and pathway for both instruments combined, whereas the percentage of task time the instrument was idle and velocity, and acceleration of the instrument were calculated individually for both, the dominant and non-dominant hands.

#### Definition of variables

##### Both instruments motion volume [cm^3^]

The motion volume is equal to a cube calculated by the respective widest motion of both instruments on the x-, y-, and z-axes.

##### Both instruments relative time out of view [%]

The sum of all times one or both instruments were out of view relative to the time per task.

##### Individual instrument’s path length [cm]

The length of the path traveled by the instrument’s tip over the entire task.

##### Individual instrument’s relative idle time [%]

The sum of all times the respective instrument was idle relative to the time per task.

##### Individual instrument’s velocity [mm/s]

The mean velocity the tip of the instrument was moved during a task.

##### Individual instrument’s acceleration [mm/s^2^]

The mean acceleration of the instrument’s tip during a task.

### Statistical analysis

Statistical analyses were performed using SPSS version 26 (IBM Corp. Armonk, NY, USA). The normality of continuous data was tested using a Kolmogorov–Smirnov test and by inspecting the frequency distributions. Participant characteristics are represented either as medians and interquartile ranges (IQRs) for continuous variables or as distributions of frequencies. Learning curves were analyzed using a repeated linear model with post-hoc correction (Bonferroni correction). No values were missing in primary analysis. For comparison between groups a Mann–Whitney-U-Test was used. The threshold for the level of significance was set at *p* < 0.05.

## Results

### Participants

The median age of participants was 23 years, and most were in their fourth year (76.7%) of medical school (Table 1). Of the participants, 34 (60.7%) were female and 22 were male (39.3%). Most participants (*n* = 50, 89.3%) were right-handed. Only 15 (26.8%) participants had previous laparoscopic experience, but none had participated in a laparoscopic training course.

**Table 1:** Basic participant characteristics

Participants	*n* (%)
Sex [*n*] (%)	
Female	34 (60.7)
Male	22 (39.3)
Median age [years] (IQR)	23 (22–24)
Handedness [*n* (%)]	
Right	50 (89.3)
Left	6 (10.7)
Year of medical school [*n*] (%)	
3rd year	6 (10.7)
4th year	43 (76.7)
5th year	7 (12.5)
Previous laparoscopic experience [*n* (%)]	15 (26.8)

### Time

The participants significantly improved their completion time per task (Table 2) for all four tasks (all tasks: *p* < 0.001). In all tasks, the students reduced their time in the final test by at least half of the baseline test.

**Table 2:** Development of the task time over the training course

	Test 1	Test 2	Test 3	Test 4	*P*-value
Peg-transfer					
Task time [sec] (IQR)	262 (210–303)	157 (130–173)	140 (122–156)	129 (108–143)	< 0.001^a^
Precision cut					
Task time (sec) (IQR)	414 (280–496)	238 (173–294)	190 (146—239)	179 (135–200)	< 0.001^a^
Balloon resection					
Task time (sec) (IQR)	422 (302–497)	250 (172–296)	222 (158–275)	209 (155–264)	< 0.001^b^
Laparoscopic suture and knot					
Task time (sec) (IQR)	641 (396–858)	362 (223–443)	259 (170–314)	244 (172–290)	< 0.001^a^

^a^Test 2 vs. Test 3 > 0.05; ^**b**^Test 2 vs. Test 3 and Test 3 vs. Test 4 > 0.05

### Both instruments’ motion volume

Motion volume was calculated for both hands combined (Table 3). The only significant change in motion volume was seen in the precision cut task, with the main reduction occurring from Test 1 to Test 4 (Test 1: 2403 cm^3^ vs. Test 4: 1422 cm^3^; *p* = 0.04). During other tasks a comparable volume (peg-transfer: Test 1: 1354 cm^3^ vs. Test 4: 1344 cm^3^; *p* = 0.37) or even a slight increase in motion volume (laparoscopic suture and knot: Test 1: 2478 cm^3^ to Test 4: 2565 cm^3^; *p* = 0.22) was observed.

**Table 3:** Development of variables for both instruments combined over the training course

	Test 1	Test 2	Test 3	Test 4	*P*-value
Peg-transfer					
Motion volume both instruments [cm^3^] (IQR)	1354 (106.3–1606)	1255 (93–1471)	1254 (92–1514)	1344 (963 – 1531)	0.37
Relative time instruments out of view [%] (IQR)	9.22 (2.78–9.33)	7.71 (0.38–7.19)	3.52 (0–3.36)	4.51 (0–3.65)	0.24
Precision cut					
Motion volume both instruments [cm^3^] (IQR)	2403 (125–263)	1644 (1042–2219)	1579 (954–2013)	1422 (22–1781)	0.011^a^
Relative time instruments out of view [%] (IQR)	13.75 (5.73–21.17)	14.43 (1.16–17.95)	7.86 (0.88–10.67)	10.87 (0.67–13.05)	0.83
Balloon resection					
Motion volume both instruments [cm^3^] (IQR)	3228 (1973–3602)	2303 (1417–2332)	2166 (1343–2184)	3054 (1345—2293)	0.29
Relative time instruments out of view [%] (IQR)	10.65 (2.34–16.12)	11.47 (9.19–15.06)	10 (1.19–12.59)	11.55 (2.25–12.71)	0.71
Laparoscopic suture and knot					
Motion volume both instruments [cm^3^] (IQR)	2478 (1618–2727)	3225 (1693–2391)	2527 (1379–2428)	2565 (1718—2567)	0.83
Relative time instruments out of view [%] (IQR)	9.83 (3.53–11.18)	6.31 (1.21–8.08)	5.73 (1.29–8.66)	5.44 (1.8–6.79)	0.22

^a^ Test 1 vs. Test 2 + 3 and Test 2 vs. Test 3 + 4 and Test 3 vs. Test 4 > 0.05

### Both instruments’ relative time out of view

Even though students reduced the relative time out of view of instruments in three out of four tasks, no significant improvement was observed in any of the tasks (Table 3). In addition, only the laparoscopic suture and knot task showed a consistent reduction in the relative time of instruments being out of view (Test 1: 9.83%, Test 2: 6.31%; Test 3: 5.73%; Test 4: 5.44%; *p* = 0.22). Both the peg-transfer and the precision cut tasks showed inconsistent development of the relative times the instruments were out of view. In the balloon resection task, the relative time instruments were out of view even increased, but was not statistically significant (Test 1: 10.65% vs. Test 4: 11.55%; *p* = 0.71). Here, instruments were out of view at least 10% of the time in all four tests.

### Individual instrument’s path length

**Fig. 2:**
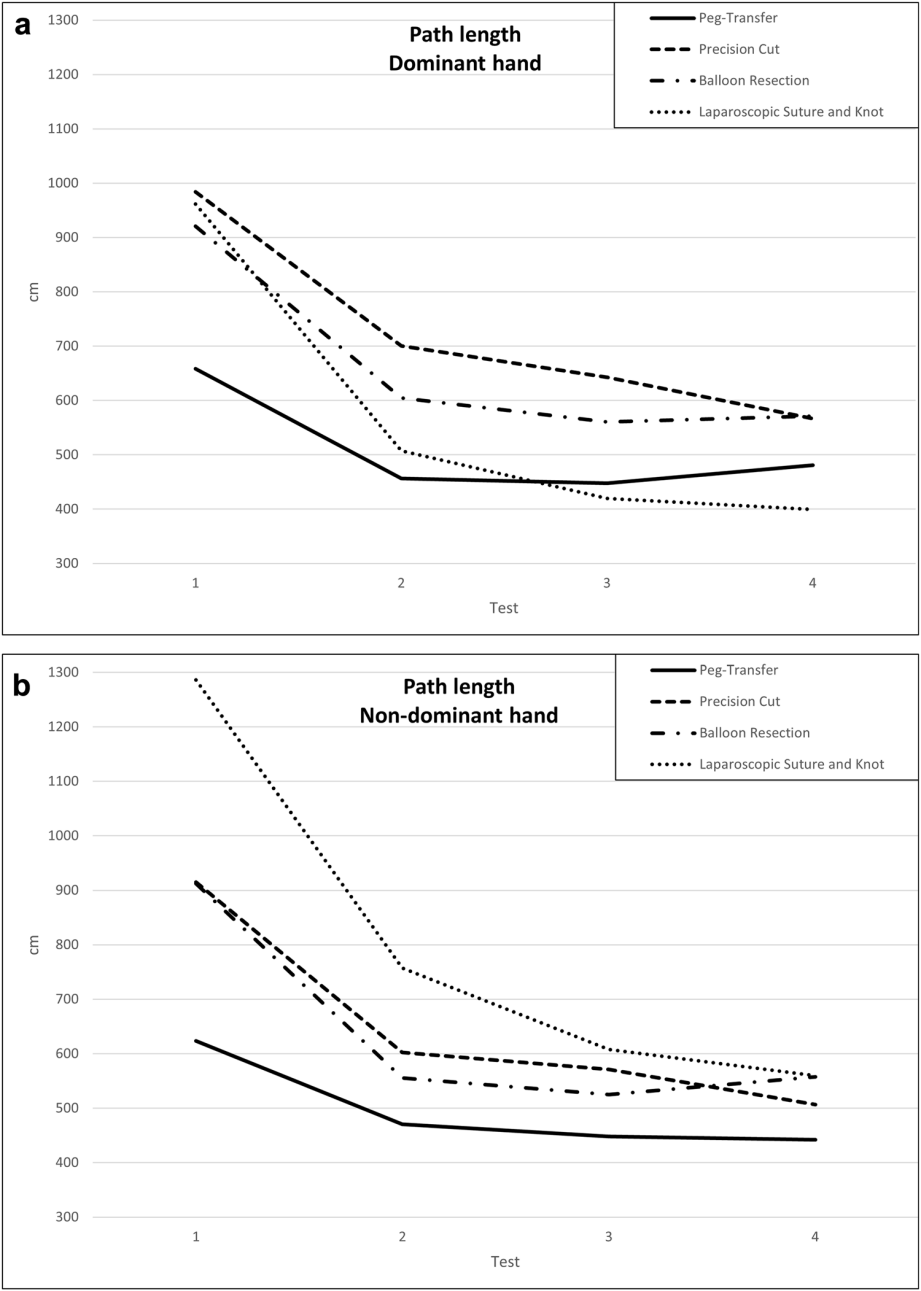
Learning curves for instrument path length for dominant (**a**) and non-dominant (**b**) hand over the course

Looking at the individual instruments we observed a significant decrease in both the non-dominant and dominant hand instrument path length in all four tasks between the first and last tests (Table 4 a–b, Fig. 2 a–b). In three out of four tasks (peg-transfer, precision cut, balloon resection) the instrument in the dominant hand showed a shorter path length than that in the non-dominant hand. Only in the second peg-transfer test and in all tests of the laparoscopic suture and knot task did the dominant hand show a higher path length.

**Table 4:** A: Development of variables for the non-dominant hand over the training course; B: Development of variables for the dominant hand over the training course

	Test 1	Test 2	Test 3	Test 4	*P*-value
**A Non-dominant hand**					
Peg-transfer					
Path length [cm] (IQR)	623.8	470.3	447.9	442.3	** < 0.001**^**a**^
	(504.8–720.1)	(386.6–533.2)	(367.8–536.8)	(367.5–484.9)	
Instrument idle time [%] (IQR)	6.56	6	5.92	5.68	** < 0.001**^**b**^
	(6.26–7.32)	(5.56–6.39)	(5.56–6.46)	(5.31–6.08)	
Mean velocity [mm/s] (IQR)	26.5	31.4	32.6	34.5	**< 0.001**^**c**^
	(23.4–29.1)	(28.8–34.4)	(28.5–35.8)	(31.4–37.6)	
Mean acceleration [mm/s^2^] (IQR)	3.09	2.06	1.87	1.8	0.18
	(1.2–3.3)	(0.98–2.86)	(1.03–2.9)	(0.71–2.49)	
Precision cut					
Path length [cm] (IQR)	915.2	602.6	571.4	506.7	** < 0.001**^**b**^
	(579.5–1144)	(332.7–812.7)	(292.8–746.9)	(281–660.2)	
Instrument idle time [%] (IQR)	6.52	6.08	6.03	5.92	> 0.05
	(5.53–7.45)	(5–7.23)	(4.52–7.75)	(4.54–7.44)	
Mean velocity [mm/s] (IQR)	26.4	28.7	32.5	31.1	> 0.05
	(20–32.5)	(18.5–36.4)	(20.6–40.1)	(20.2–39.6)	
Mean acceleration [mm/s^2^] (IQR)	3.73	4.28	3.95	3.66	0.66
	(1.76–4.51)	(1.59–4.93)	(1.25–6.11)	(1.41–4.73)	
Balloon resection					
Path length [cm] (IQR)	912.1	555.3	524.7	557.2	** < 0.001**^**a**^
	(630.8–1083.4)	(386.1–674.5)	(360.3–620.2)	(337.1–663.3)	
Instrument idle time [%] (IQR)	7.14	6.74	6.6	6.39	0.24
	(6.62–7.77)	(6.31–7.66)	(6.11–7.51)	(5.87–7.2)	
Mean velocity [mm/s] (IQR)	24.2	29.3	27.9	27.9	** < 0.01**^**f**^
	(20.3–27.7)	(21.8–30)	(23.3–33.6)	(23.2–31.8)	
Mean acceleration [mm/s^2^] (IQR)	3.55	4.44	6.42	5.16	0.09
	(1.68–4.52)	(1.87–5.23)	(1.95–6.78)	(2.25–5.77)	
Laparoscopic suture and knot					
Path length [cm] (IQR)	1286	757.5	607.8	559.6	** < 0.001**^**a**^
	(658.1–1649.4)	(427.1–935.7)	(324.3–667.3)	(346.6–680.2)	
Instrument idle time [%] (IQR)	7.01	7.04	6.76	6.65	0.14
	(6.54–7.51)	(6.47–7.59)	(6.24–7.26)	(6.15–7.28)	
Mean velocity [mm/s] (IQR)	24.9	27.2	29.3	28.5	**< 0.01**^d^
	(21.12–27.8)	(21.5–27.7)	(23.2–30.3)	(24.3–32.7)	
Mean acceleration [mm/s^2^] (IQR)	5.64	4.93	6.54	7.58	0.11
	(3.17–6.35)	(2.7–7.09)	(3.65–9.03)	(4.28–8.7)	
**B Dominant hand**					
Peg-transfer					
Path length [cm] (IQR)	658.3	456.7	448	480.7	** < 0.001**^**a**^
	(517.8–778.4)	(389–519.2)	(378.3–497.7)	(357.9–509.3)	
Instrument idle time [%] (IQR)	6.67	5.75	5.67	5.07	** < 0.001**^**b**^
	(6.11–7.24)	(5.28–6.52)	(5.08–6.37)	(4.43–5.91)	
Mean velocity [mm/s] (IQR)	26.7	31.3	33.4	38.6	** < 0.001**
	(24.1–28.8)	(28.4–34.2)	29.3–37.1)	(31.4–43.2)	
Mean acceleration [mm/s^2^] (IQR)	3.15	2.75	2.51	4.5	0.06
	(1.2–3.74)	(0.95–3.15)	(1.5–3.89)	(1.45–5.99)	
Precision cut					
Path length [cm] (IQR)	984.2	700.3	642.4	566.5	** < 0.001**^**c**^
	(648–1185.3)	(456.8–876.2)	(445.2–780.4)	(424–668.8)	
Instrument idle time [%] (IQR)	6.33	5.75	5.28	5.54	** < 0.001**^**d**^
	(5.74–7.04)	(5.31–6.49)	(4.69–6.1)	(4.7–6.5)	
Mean velocity [mm/s] (IQR)	28.8	32.7	36.5	35	** < 0.001**^e^
	(24.4–32.3)	(28.9–35.6)	(30.8–40.9)	(29.1–40.8)	
Mean acceleration [mm/s^2^] (IQR)	4.27	3.73	3.33	3.4	0.5
	(2.08–6.19)	(1.49–5.31)	(1.11–4.84)	(1.41–4.95)	
Balloon resection					
Path length [cm] (IQR)	920.8	604.7	560.4	571.5	** < 0.001**^**a**^
	(646.2–1038.3)	(393.9–707.6)	(350.7–634.8)	(355.4–660.7)	
Instrument idle time [%] (IQR)	7.17	7.13	7.07	6.9	0.18
	(6.54–7.81)	(6.79–7.6)	(6.56–7.68)	(6.26–7.73)	
Mean velocity [mm/s] (IQR)	24.5	28.9	26.7	27.7	** < 0.01**^f^
	(20.1–27.7)	(21.7–28.6)	22.4–29.7)	(22.2–32.4)	
Mean acceleration [mm/s^2^] (IQR)	3.62	2.53	3.58	3.47	0.78
	(1.89–4.34)	(1.15–3.33)	(1.87–5.06)	(1.52–5.05)	
Laparoscopic suture and knot					
Path length [cm] (IQR)	961.3	507.3	419.7	399.6	** < 0.001**^**a**^
	(469–1369.9)	(267.2–656.3)	(202.5–648.2)	(237.3–522.7)	
Instrument idle time [%] (IQR)	6.41	6.45	6.13	6.05	0.1
	(6.11–7.13)	(5.91–7.06)	(5.66–6.93)	(5.54–6.67)	
Mean velocity [mm/s] (IQR)	27.9	33.6	31.4	33	** < 0.01**^ g^
	(25–30.8)	(25.1–34.4)	(26.8–34.6)	(28.5–36.4)	
Mean acceleration [mm/s^2^] (IQR)	13.42	12.75	14.88	16.32	0.35
	(9.59–19.56)	(8.38–17.94)	(8.8–16.08)	(9.64–18.59)	

Significant *P*-values are marked in bold

A ^a^Test 2 vs. Test 3 + Test 4 > 0.05 and Test 3 vs. Test 4 > 0.05; ^b^Test 2 vs. Test 3 and Test 3 vs. Test 4 > 0.05; ^c^ Test 2 vs. Test 3 > 0.05; ^d^Test 1 vs. Test 2 + 3 and Test 2 vs. Test 3 + 4 and Test 3 vs. Test 4 > 0.05

B ^a^Test 2 vs. Test 3 + Test 4 > 0.05 and Test 3 vs. Test 4 > 0.05;^b^Test 2 vs. Test 3 > 0.05; ^c^Test 2 vs. Test 3 and Test 3 vs. Test 4 > 0.05; ^d^ Test 2 vs. Test 4 > 0.05; ^e^Test 2 vs. Test 4 and Test 3 vs. Test 4 > 0.05; ^f^Test 1 vs. Test 3 + 4 and Test 2 vs. Test 3 + 4 and Test 3 vs. Test 4 > 0.05; ^g^Test 1 vs. Test 2 + 3 and Test 2 vs. Test 3 + 4 and Test 3 vs. Test 4 > 0.05

### Individual instrument’s velocity

**Fig. 3:**
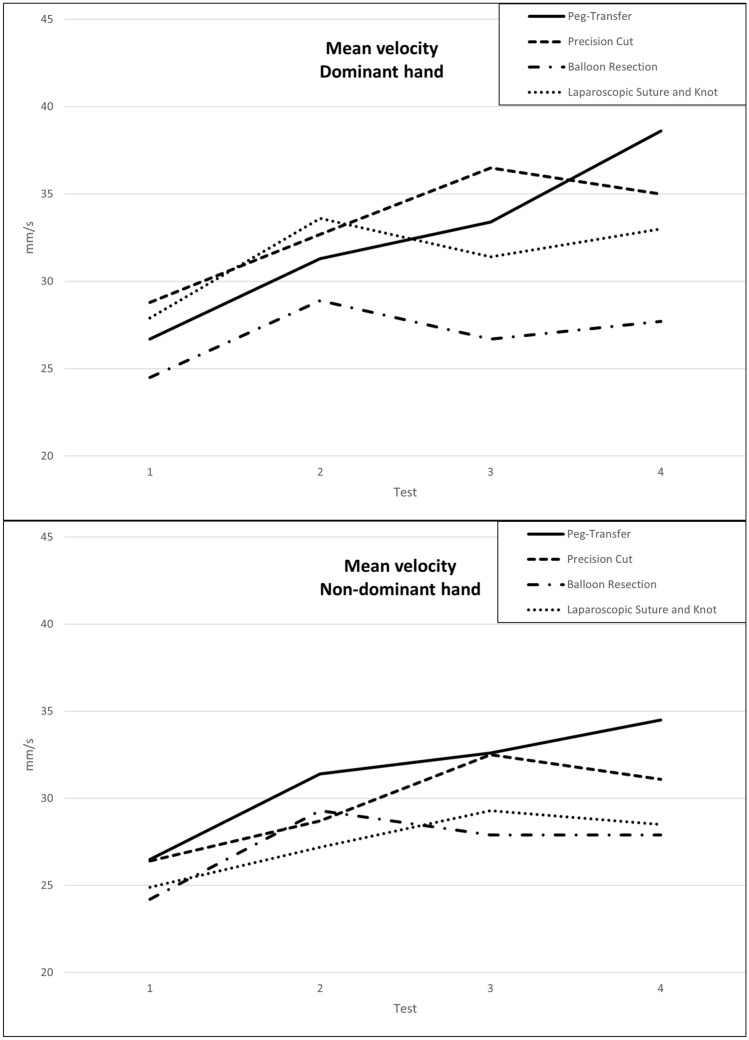
Learning curves for instrument mean velocity for dominant (**a**) and non-dominant (**b**) hand over the course

We observed that the participants became significantly faster with both their dominant and non-dominant hands in three out of four tasks (Table 4 a–b, Fig. 3 a–b). In the peg-transfer task, participants showed the highest velocities for both hands (Test 4 non-dominant: 34.5 mm/s vs. Test 4 dominant hand: 38.6 mm/s), respectively. Whereas the dominant hand became significantly faster in the precision-cut task (dominant hand: *p* < 0.001), but the non-dominant instrument did not improve significantly.

### Individual instrument’s relative idle time

**Fig. 4:**
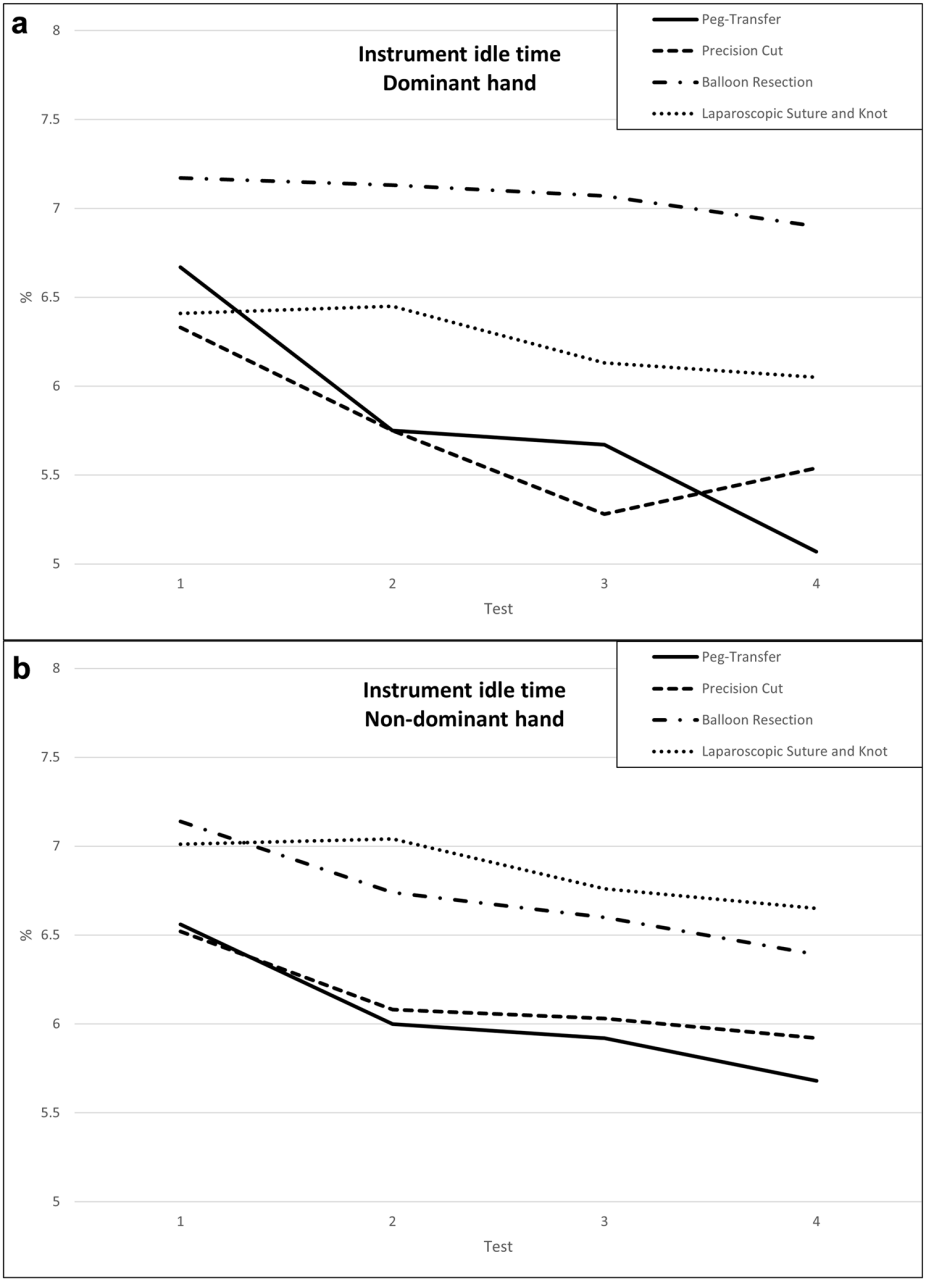
Learning curves for instrument idle time for dominant (**a**) and non-dominant (**b**) hand over the course

In the peg-transfer task, a significant reduction in the relative instrument idle time was observed for both hands, whereas in the precision cut task, only the instrument in the dominant hand showed a significant reduction in idle time (Table 4 a–b, Fig. 4 a–b). In the laparoscopic suture and knot and balloon resection tasks, the relative idle times of both instruments did not differ significantly between the dates of data collection.

### Individual instrument’s acceleration

**Fig. 5:**
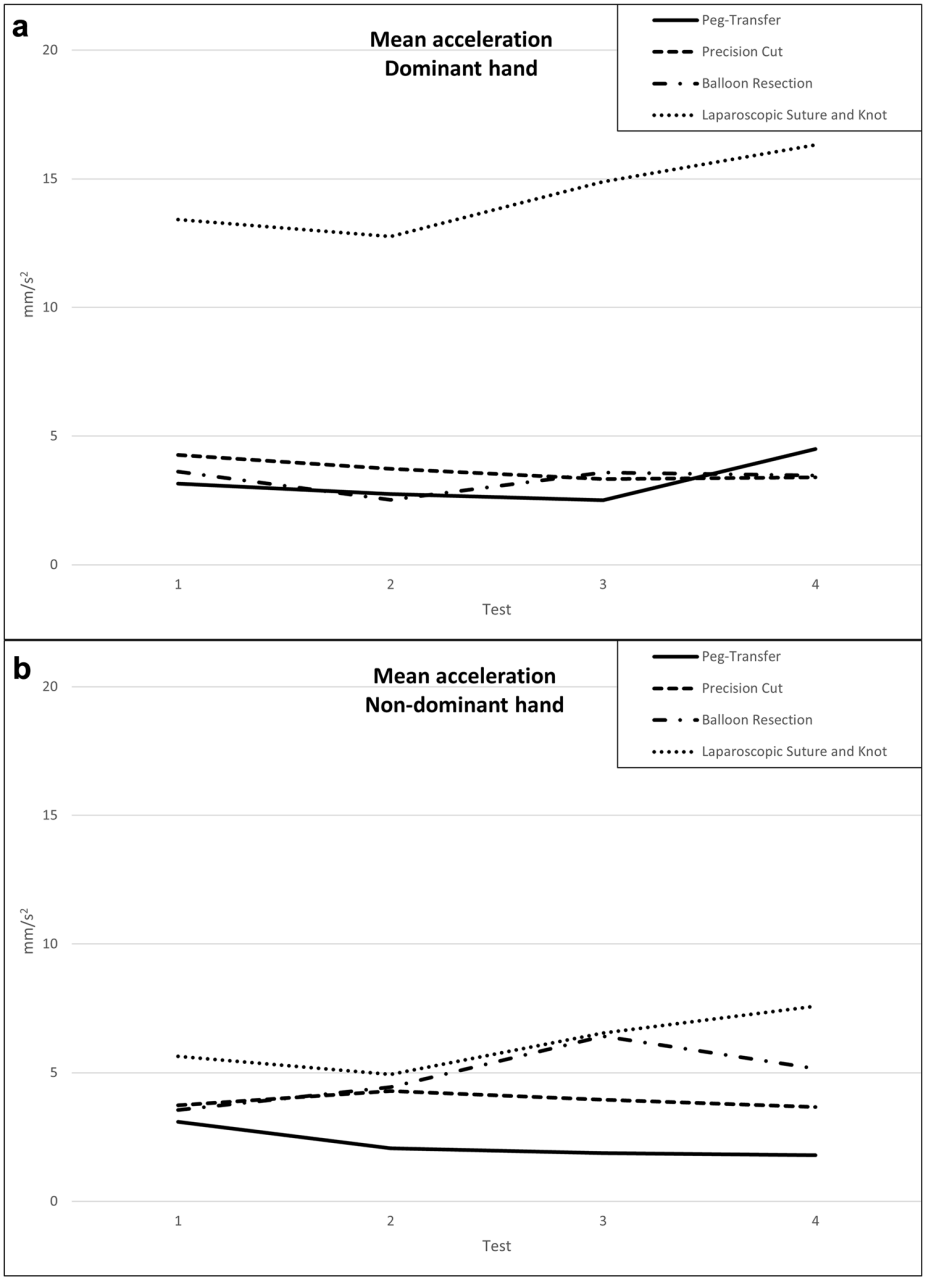
Learning curves for instrument mean acceleration for dominant (**a**) and non-dominant (**b**) hand over the course

The trend for instrument acceleration was inconclusive for either the dominant or non-dominant hand (Table 4 a*–*b, Fig. 5 a*–*b). No significant change was observed in any of the tasks.

### Comparison between participants with and without previous laparoscopic experience

The comparison between participants without (*n* = 41) and with previous laparoscopic experience (*n* = 15) showed mostly no differences. However, in the first (baseline) test of the Precision cut task participants showed significant differences in path length of the dominant hand (no experience: 9491.8 cm vs. experience: 6245.7 cm; *p* = 0.044) and the non-dominant hand (no experience: 10,068.7 cm vs. experience: 6579.6 cm; *p* = 0.036) (Supplementary Table. 1). The non-dominant hand’s path length remained significant shorter for the experienced group during Precision cut task in the second test (no experience: 6662.7 cm vs. experience: 4685.1 cm; *p* = 0.027) (Supplementary Table 2). Similarly, in the Laparoscopic suture and knot task there was a significant difference of the non-dominant hand’s path length in test 3 (no experience: 4186.9 cm vs. experience: 2002.7 cm; *p* = 0.042) (Supplementary Material 3). For the same task and test there was also a significant difference in the relative time of instruments being out of view in favor of the group with no experience (no experience: 2% vs. experience: 14%; *p* = 0.002). In the final test, participants with previous laparoscopic experience showed significant better acceleration compared to participants without experience (no experience: 2.7 mm/s^2^ vs. experience: 4.4 mm/s^2^; *p* = 0.025) (Supplementary Material 4).

## Discussion

This prospective study aimed to investigate the learning curves of minimally invasive surgical skills developed during a standardized training course. For instrument motion analysis, a novel and objective measurement tool was employed to assess changes in complex motion parameters over the course of a standardized training curriculum. Thus, the data allow for a more detailed investigation of the laparoscopic learning curve in general and for both hands individually. Furthermore, certain parameters, such as the relative time of instruments being out of view and volume of motion, both surrogate parameters for safety and precision, were computed and investigated. A high motion volume and time of instruments being out of view are signs of wide movements, which could potentially cause damage to nearby tissues, as discussed by Hardon et al.[15]

Most parameters showed a significant learning curve, usually with the most pronounced improvement between the first (baseline) and second measurements. This indicates a rapid and steep learning curve. However, the learning curve of instrument velocity revealed an interesting pattern. Except for the peg-transfer task, in which the subjects became faster across all tests, the other tasks showed the fastest time in Test 2 or 3, and finally a slightly decreased velocity in Test 4. This could be an indication that the efficient execution of a task depends less on speed, but on the improvement of other parameters, for example, a reduced path length or less instrument idle time. Sufficient interaction of various skills could enhance efficiency. This hypothesis was supported by the continuous improvement in task time, with the best task times in Test 4. However, improvements in other parameters, such as acceleration or idle time, even though some of them did not significantly change in our trial, might also play an important role in improving efficiency and time per task.

The steep improvement in task time and path length were similarly described in another study by Hardon et al. In contrast to our approach, this study used a continuous measurement of each repetition [15]. Even in more complex training scenarios, only a few sessions were necessary to observe a relevant and significant improvement in procedure time and skills [19].

An additional finding of our trial was that the dominant and non-dominant hands improved significantly with the same parameters. In the precision cut task, the dominant hand showed significant improvements in idle time and velocity, while the non-dominant hand did not. However, a direct comparison between the hands is not useful because most exercises are not designed to use and train both hands equally. Peg-transfer may be the only exception since both hands are used equally in the optimum case. In fact, the values for path length, idle time, and velocity were in a comparable range, but on average the dominant hand was moved slightly faster, further, and more frequently. This may indicate that the learning effect in novices is more pronounced for the dominant hand. Another study, which also examined differences between instrument movements with the dominant and non-dominant hand, respectively, showed an improved smoothness of the dominant hand, especially in experienced surgeons [20].

Furthermore, we analyzed two variables that were considered relevant to surgical safety: instruments out of view and the volume of instrument motion. Except for the precision cut task, there was no improvement in the volume of instrument motion. Regarding the percentage of time the instruments were out of view, no significant improvement was observed in any of the tasks. Although the training focused specifically on avoiding such behavior, the participants did not improve in this regard. This is a very interesting finding because even in the last test, the subjects moved the instruments out of view between 4.5% and 11.5% of the task time. However, a comparison with the existing literature was not possible, since we did not find any other trial investigating the movements of instruments outside the field of endoscopic view.

The non-significant improvement in movement volume could indicate that the training curriculum was not designed for this purpose, but could also be indicative of a significantly longer learning curve. The latter was concluded by Kunert et al., who stated that efficiency was learned faster than precision [21]. A potential measure to increase precision could be the regular use of video tutorials during the training course [22]. In general, it seems that safety and precision parameters are often under-reported, but should be given a higher priority, even over task time as demanded by von Websky et al.[23]

Comparing participants with and without laparoscopic experience showed almost no differences indicating that both groups were comparable in most instances. In this comparison, the results are inconclusive and do not indicate a stronger effect of prior laparoscopic experience. For some significant results, a false-positive finding (Type II error) must also be considered due to the high number of statistical tests. The only systematic significant differences were found in the path length of the nondominant hand and only in the first three tests. A possible conclusion would be that participants with previous laparoscopic experience have a slight advantage in terms of motion efficiency. However, with the performance of both groups equalizing again at the end of the training, it can be assumed that a comparable plateau level was reached. This is to some extent consistent with the observation of Hardon et al. who described that a plateau phase in terms of path length was reached after only a few task repetitions [15].

### Strengths and limitations

To the best of our knowledge, this study is the first to describe the motion parameters and learning curves of both hands individually. In addition, the investigation of safety-related parameters, such as motion volume and instruments being out of view has not yet been discussed regarding learning curves. Another quality of our trial was the use of the IMA tool. This device allows the objective measurement of various motion variables in a box-trainer setting [16]. Comparable qualitative variables can usually only be measured using VR-simulators. However, the latter lacks a certain degree of realism, for example regarding the handling of instruments and haptic feedback [24]. However, since there was only one device fitted with the IMA, a continuous measurement of each repetition for each participant was not feasible. Consequently, our learning curve consisted of only four different testing time points. In comparison, other studies were able to provide continuous measurement, but lacked either participants or the number of tasks examined [15, 21].

## Conclusion

Our results show that an FLS-based and modified training curriculum successfully increases the motion efficiency of participants by increasing speed and reducing idle time and path length. However, the training course failed to improve parameters relevant to surgical precision and safety, such as the volume of motion and relative time of instruments being out of view. Most training curricula are designed and validated by efficiency parameters. Thus, surgical quality and safety should be given greater consideration and incorporated into basic skills training accordingly.

## Supplementary Information

Below is the link to the electronic supplementary material.

Supplementary file1 (DOCX 46 KB)

## Data Availability

The participants of this study did not give written consent for their data to be shared publicly, so due to the sensitive nature of the research supporting data is not available.
