# Supplementary material for: Does practice make perfect? Laparoscopic training mainly improves motion efficiency: a prospective trial
Source: Updates Surg. 2023 May 9;75(5):1103–15. doi: 10.1007/s13304-023-01511-w (PMC10359367; doi:10.1007/s13304-023-01511-w)
Supplement: Supplementary file 1 — Supplementary file1 (DOCX 46 KB) [file 13304_2023_1511_MOESM1_ESM.docx]

|  |  | **Test 1** | | |
| --- | --- | --- | --- | --- |
|  |  | **previous laparoscopic  experience** | **no laparoscopic  experience** | **p-value** |
|  |  | Median (IQR) | Median (IQR) |  |
| **Peg-Transfer** | |  |  |  |
|  | Task time [sec] | 223.6 (202.2 - 298.8) | 250 (213.3 - 307.1) | 0.259 |
|  | Motion volume both instruments [cm³] | 1148 (1035 - 1530) | 1302 (1064 - 1670) | 0.518 |
|  | Relative time instruments out of view [%] | 4.6 (2.9 - 9.1) | 5.2 (2.7 - 10.7) | 0.678 |
|  | Dominant hand Path length [cm] | 5722.5 (4655.1 - 7925.6) | 6067.8 (5137.6 - 7196.8) | 0.622 |
|  | Dominant hand Idle time [%] | 0.7 (0.6 - 0.7) | 0.7 (0.6 - 0.7) | 0.692 |
|  | Dominant hand Mean velociy [mm/s] | 27.3 (23.5 - 29.2) | 25.8 (23.2 - 28.4) | 0.692 |
|  | Dominant hand Mean acceleration [mm/s^2^] | 2.6 (1.5 - 3.9) | 1.9 (1.2 - 3) | 0.173 |
|  | Non-dominant hand Path length [cm] | 5640.2 (4606.4 - 6696.1) | 6198.1 (5251.5 - 7796.3) | 0.259 |
|  | Non-dominant hand Idle time [%] | 0.7 (0.6 - 0.7) | 0.7 (0.6 - 0.7) | 0.582 |
|  | Non-dominant hand Mean velociy [mm/s] | 26.9 (24.3 - 28.8) | 25.7 (24.1 - 28.9) | 0.636 |
|  | Non-dominant hand Mean acceleration [mm/^2^] | 1.5 (1 - 3.7) | 1.7 (1.3 - 3.8) | 0.779 |
| **Precision Cut** | |  |  |  |
|  | Task time [sec] | 321.1 (234.9 - 443.3) | 411.3 (288.7 - 550.7) | 0.063 |
|  | Motion volume both instruments [cm³] | 1435 (720 - 2608) | 1728 (1271 - 2697) | 0.569 |
|  | Relative time instruments out of view [%] | 9 (3.3 - 22.4) | 11.5 (7.4 - 21.1) | 0.582 |
|  | Dominant hand Path length [cm] | 6245.7 (5175.2 - 8441.9) | 9491.8 (6925.6 - 12141.4) | **0.044** |
|  | Dominant hand Idle time [%] | 0.7 (0.6 - 0.7) | 0.7 (0.5 - 0.7) | 0.609 |
|  | Dominant hand Mean velociy [mm/s] | 23.9 (20.1 - 32.4) | 26 (19.9 - 33.1) | 0.779 |
|  | Dominant hand Mean acceleration [mm/s^2^] | 3.2 (1.6 - 4.6) | 2.9 (1.9 - 4.3) | 0.794 |
|  | Non-dominant hand Path length [cm] | 6579.6 (5930.1 - 10170.8) | 10068.7 (6942.2 - 13758.3) | **0.036** |
|  | Non-dominant hand Idle time [%] | 0.6 (0.6 - 0.7) | 0.6 (0.6 - 0.7) | 0.543 |
|  | Non-dominant hand Mean velociy [mm/s] | 28.3 (22.4 - 31.5) | 28.5 (25.6 - 32.5) | 0.505 |
|  | Non-dominant hand Mean acceleration [mm/^2^] | 3.2 (1.7 - 6.2) | 3.1 (2.3 - 6.2) | 0.493 |
| **Ballon Resection** | |  |  |  |
|  | Task time [sec] | 398.8 (303.8 - 433.6) | 399.4 (288.5 - 571.8) | 0.505 |
|  | Motion volume both instruments [cm³] | 2548 (2207 - 2890) | 2669 (1859 - 3977) | 0.721 |
|  | Relative time instruments out of view [%] | 7.6 (2.4 - 16.5) | 8 (2.3 - 16.1) | 0.779 |
|  | Dominant hand Path length [cm] | 8151.9 (5709.9 - 9050.2) | 8209.9 (6408 - 11624) | 0.481 |
|  | Dominant hand Idle time [%] | 0.7 (0.7 - 0.8) | 0.7 (0.7 - 0.8) | 0.931 |
|  | Dominant hand Mean velociy [mm/s] | 23 (20 - 29.8) | 23.1 (20.4 - 27.2) | 0.915 |
|  | Dominant hand Mean acceleration [mm/s^2^] | 4.5 (2 - 6.4) | 2.9 (1.7 - 4.3) | 0.161 |
|  | Non-dominant hand Path length [cm] | 7792.3 (6490.1 - 9962.2) | 8605.7 (6379.5 - 12790.6) | 0.423 |
|  | Non-dominant hand Idle time [%] | 0.7 (0.7 - 0.8) | 0.7 (0.6 - 0.8) | 0.259 |
|  | Non-dominant hand Mean velociy [mm/s] | 24 (18.8 - 26.5) | 25.3 (20.5 - 29.3) | 0.22 |
|  | Non-dominant hand Mean acceleration [mm/^2^] | 2.8 (1.3 - 4.6) | 3.3 (1.9 - 4.1) | 0.992 |
| **Laparoscopic Suture and Knot** | |  |  |  |
|  | Task time [sec] | 550.6 (373.7 - 850.6) | 554.3 (417.4 - 878.9) | 0.493 |
|  | Motion volume both instruments [cm³] | 1888 (1548 - 2770) | 2168 (1618 - 2721) | 0.635 |
|  | Relative time instruments out of view [%] | 5.1 (2.6 - 10.4) | 7 (3.9 - 12.7) | 0.369 |
|  | Dominant hand Path length [cm] | 7265.8 (5804.1 - 22242.5) | 10978.3 (7359.3 - 16375.4) | 0.609 |
|  | Dominant hand Idle time [%] | 0.7 (0.7 - 0.7) | 0.7 (0.7 - 0.8) | 0.22 |
|  | Dominant hand Mean velociy [mm/s] | 26.2 (23.2 - 29.2) | 23.8 (20.9 - 27.7) | 0.243 |
|  | Dominant hand Mean acceleration [mm/s^2^] | 5.2 (3.9 - 7.1) | 4.5 (3 - 6.2) | 0.199 |
|  | Non-dominant hand Path length [cm] | 10629.1 (4677.9 - 13616.8) | 7673.6 (4590.7 - 14742.4) | 0.864 |
|  | Non-dominant hand Idle time [%] | 0.7 (0.6 - 0.7) | 0.7 (0.5 - 0.7) | 0.166 |
|  | Non-dominant hand Mean velociy [mm/s] | 26.8 (24.3 - 28.8) | 27.9 (25.2 - 31.1) | 0.343 |
|  | Non-dominant hand Mean acceleration [mm/^2^] | 13.6 (10.2 - 23.9) | 11.7 (8.4 - 19.3) | 0.441 |

Supplementary Table 1: Comparison of test results (Test 1) between participants with previous laparoscopic experience vs. participants with no laparoscopic experience (significant p-values markes bold)

|  |  | **Test 2** | | |
| --- | --- | --- | --- | --- |
|  |  | **previous laparoscopic  experience** | **no laparoscopic  experience** | **p-value** |
|  |  | Median (IQR) | Median (IQR) |  |
| **Peg-Transfer** | |  |  |  |
|  | Task time [sec] | 135.2 (128 - 167.2) | 155.3 (139.2 - 182.6) | 0.136 |
|  | Motion volume both instruments [cm³] | 1122 (880 - 1319) | 1225 (938 - 1529) | 0.263 |
|  | Relative time instruments out of view [%] | 0.6 (0.3 - 5.8) | 2.5 (0.5 - 7.7) | 0.221 |
|  | Dominant hand Path length [cm] | 4309.7 (3579.6 - 5372.1) | 4545.6 (3931.9 - 5294.1) | 0.65 |
|  | Dominant hand Idle time [%] | 0.6 (0.5 - 0.6) | 0.6 (0.6 - 0.6) | 0.817 |
|  | Dominant hand Mean velociy [mm/s] | 31.9 (28.8 - 35.1) | 30.2 (28.7 - 34.4) | 0.718 |
|  | Dominant hand Mean acceleration [mm/s^2^] | 1.2 (0.7 - 2.3) | 2.1 (1.1 - 3.3) | 0.087 |
|  | Non-dominant hand Path length [cm] | 4386.2 (3860.1 - 5204.3) | 4476.1 (3940 - 5206.3) | 0.677 |
|  | Non-dominant hand Idle time [%] | 0.6 (0.5 - 0.7) | 0.6 (0.5 - 0.6) | 0.637 |
|  | Non-dominant hand Mean velociy [mm/s] | 31.4 (28.7 - 34.9) | 30.7 (28.2 - 33.3) | 0.523 |
|  | Non-dominant hand Mean acceleration [mm/^2^] | 2.4 (0.9 - 4.2) | 1.6 (1 - 3.1) | 0.454 |
| **Precision Cut** | |  |  |  |
|  | Task time [sec] | 176 (149.4 - 252.9) | 233.3 (182.2 - 304.8) | 0.058 |
|  | Motion volume both instruments [cm³] | 1651 (906 - 2268) | 1390 (1067 - 2118) | 0.993 |
|  | Relative time instruments out of view [%] | 13.2 (0.9 - 31.7) | 7.9 (1.2 - 17.1) | 0.488 |
|  | Dominant hand Path length [cm] | 4698.1 (2054.5 - 5595.9) | 5752.5 (3787.2 - 8759.2) | 0.058 |
|  | Dominant hand Idle time [%] | 0.5 (0.5 - 0.6) | 0.6 (0.5 - 0.8) | 0.094 |
|  | Dominant hand Mean velociy [mm/s] | 34.2 (17.6 - 36.4) | 28.6 (18.7 - 36.3) | 0.637 |
|  | Dominant hand Mean acceleration [mm/s^2^] | 3.2 (1.5 - 6) | 2.6 (1.7 - 4.4) | 0.296 |
|  | Non-dominant hand Path length [cm] | 4685.1 (4199.4 - 6429.9) | 6662.7 (5067.3 - 9342) | **0.027** |
|  | Non-dominant hand Idle time [%] | 0.6 (0.5 - 0.6) | 0.6 (0.5 - 0.7) | 0.875 |
|  | Non-dominant hand Mean velociy [mm/s] | 32.8 (29.9 - 35.1) | 31.7 (28.8 - 36) | 0.598 |
|  | Non-dominant hand Mean acceleration [mm/^2^] | 2.7 (1.5 - 4.8) | 2.5 (1.4 - 5.5) | 0.846 |
| **Ballon Resection** | |  |  |  |
|  | Task time [sec] | 209.1 (163.7 - 305.7) | 208.6 (172.8 - 294.3) | 0.817 |
|  | Motion volume both instruments [cm³] | 1660 (1414 - 2070) | 1832 (1398 - 2385) | 0.443 |
|  | Relative time instruments out of view [%] | 7.7 (1.2 - 29.5) | 2.9 (0.8 - 14.1) | 0.494 |
|  | Dominant hand Path length [cm] | 5164.8 (4212.2 - 6372.3) | 5575.7 (3831.5 - 7084.5) | 0.664 |
|  | Dominant hand Idle time [%] | 0.7 (0.6 - 0.8) | 0.7 (0.6 - 0.8) | 0.548 |
|  | Dominant hand Mean velociy [mm/s] | 26 (21.7 - 32.2) | 25.1 (21.8 - 29.9) | 0.963 |
|  | Dominant hand Mean acceleration [mm/s^2^] | 3.4 (1.6 - 5.7) | 3.2 (1.9 - 5.1) | 0.948 |
|  | Non-dominant hand Path length [cm] | 5168.4 (3486.8 - 6295.3) | 5358.8 (4099.1 - 7186.7) | 0.443 |
|  | Non-dominant hand Idle time [%] | 0.7 (0.7 - 0.8) | 0.7 (0.7 - 0.8) | 0.535 |
|  | Non-dominant hand Mean velociy [mm/s] | 24.3 (22.4 - 26.6) | 24.9 (21.6 - 29.1) | 0.677 |
|  | Non-dominant hand Mean acceleration [mm/^2^] | 2.2 (1.1 - 5.4) | 2.1 (1.2 - 3.3) | 0.774 |
| **Laparoscopic Suture and Knot** | |  |  |  |
|  | Task time [sec] | 256.7 (225.3 - 392.6) | 324.3 (218.8 - 466.2) | 0.65 |
|  | Motion volume both instruments [cm³] | 1996 (1904 - 2276) | 1941 (1615 - 2475) | 0.732 |
|  | Relative time instruments out of view [%] | 2 (0.5 - 8.1) | 2.9 (1.4 - 8.2) | 0.215 |
|  | Dominant hand Path length [cm] | 6234.2 (4222 - 10017.3) | 5995.9 (4497.4 - 9348.1) | 0.803 |
|  | Dominant hand Idle time [%] | 0.7 (0.6 - 0.8) | 0.7 (0.6 - 0.8) | 0.56 |
|  | Dominant hand Mean velociy [mm/s] | 24.4 (19.7 - 27.2) | 24.5 (22 - 28) | 0.788 |
|  | Dominant hand Mean acceleration [mm/s^2^] | 2.9 (2.1 - 7) | 5 (3 - 7.1) | 0.35 |
|  | Non-dominant hand Path length [cm] | 4361.8 (3413.7 - 6229.8) | 3932.1 (1938 - 7147.5) | 0.39 |
|  | Non-dominant hand Idle time [%] | 0.6 (0.6 - 0.7) | 0.6 (0.6 - 0.7) | 0.89 |
|  | Non-dominant hand Mean velociy [mm/s] | 29.7 (24.5 - 30.7) | 29.3 (25.3 - 34.8) | 0.598 |
|  | Non-dominant hand Mean acceleration [mm/^2^] | 12.7 (7 - 14.7) | 12.8 (8.6 - 27) | 0.296 |

Supplementary Table 2: Comparison of test results (Test 2) between participants with previous laparoscopic experience vs. participants with no laparoscopic experience (significant p-values markes bold)

|  |  | **Test 3** | | |
| --- | --- | --- | --- | --- |
|  |  | **previous laparoscopic  experience** | **no laparoscopic  experience** | **p-value** |
|  |  | Median (IQR) | Median (IQR) |  |
| **Peg-Transfer** | |  |  |  |
|  | Task time [sec] | 135.2 (114.1 - 147) | 138.9 (125.2 - 160.7) | 0.153 |
|  | Motion volume both instruments [cm³] | 1222 (914 - 1643) | 1166 (931 - 1506) | 0.643 |
|  | Relative time instruments out of view [%] | 1 (0 - 6) | 1 (0 - 3) | 0.564 |
|  | Dominant hand Path length [cm] | 4110.9 (3716.5 - 5388.1) | 4099.1 (3641.9 - 5265.5) | 0.938 |
|  | Dominant hand Idle time [%] | 0.6 (0.5 - 0.6) | 0.6 (0.6 - 0.7) | 0.137 |
|  | Dominant hand Mean velociy [mm/s] | 33.6 (30.9 - 36.3) | 31.5 (28.3 - 35.4) | 0.137 |
|  | Dominant hand Mean acceleration [mm/s^2^] | 1.4 (0.6 - 2.5) | 1.9 (1.1 - 3.5) | 0.164 |
|  | Non-dominant hand Path length [cm] | 4193.2 (3777.5 - 4833.6) | 4384.4 (3725.6 - 5232.8) | 0.511 |
|  | Non-dominant hand Idle time [%] | 0.6 (0.5 - 0.6) | 0.6 (0.5 - 0.6) | 0.735 |
|  | Non-dominant hand Mean velociy [mm/s] | 32.5 (29.7 - 36.8) | 32 (29.2 - 37.6) | 0.832 |
|  | Non-dominant hand Mean acceleration [mm/^2^] | 1.7 (1.5 - 2.8) | 2.1 (1.5 - 4.1) | 0.44 |
| **Precision Cut** | |  |  |  |
|  | Task time [sec] | 160.5 (126.8 - 175.4) | 193.9 (148.5 - 248.1) | 0.066 |
|  | Motion volume both instruments [cm³] | 1317 (897 - 2295) | 1416 (956 - 1973) | 0.923 |
|  | Relative time instruments out of view [%] | 0 (0 - 0.1) | 0 (0 - 0.1) | 0.948 |
|  | Dominant hand Path length [cm] | 4902.3 (2272.3 - 6199.4) | 5299.4 (3162.4 - 8191.4) | 0.216 |
|  | Dominant hand Idle time [%] | 0.6 (0.5 - 0.8) | 0.6 (0.4 - 0.8) | 0.985 |
|  | Dominant hand Mean velociy [mm/s] | 33.6 (22.8 - 41.9) | 35.3 (19.8 - 40) | 0.877 |
|  | Dominant hand Mean acceleration [mm/s^2^] | 3.7 (1.2 - 9.6) | 2.8 (1.2 - 5.6) | 0.354 |
|  | Non-dominant hand Path length [cm] | 4867.3 (4122.8 - 6098) | 6044.2 (4508.2 - 8766.4) | 0.072 |
|  | Non-dominant hand Idle time [%] | 0.5 (0.5 - 0.6) | 0.5 (0.5 - 0.6) | 0.524 |
|  | Non-dominant hand Mean velociy [mm/s] | 35.4 (30.8 - 40.2) | 36.1 (30.8 - 41.4) | 0.657 |
|  | Non-dominant hand Mean acceleration [mm/^2^] | 3.1 (1.5 - 6.4) | 2.6 (1 - 4.5) | 0.354 |
| **Ballon Resection** | |  |  |  |
|  | Task time [sec] | 223.1 (162 - 274.5) | 197.8 (139.1 - 276.6) | 0.575 |
|  | Motion volume both instruments [cm³] | 1933 (1517 - 3336) | 1715 (1276 - 2119) | 0.189 |
|  | Relative time instruments out of view [%] | 3 (1 - 20) | 3 (1 - 10.5) | 0.372 |
|  | Dominant hand Path length [cm] | 4775.7 (3753.3 - 6241.7) | 4405.3 (3583 - 6434.7) | 0.629 |
|  | Dominant hand Idle time [%] | 0.7 (0.6 - 0.7) | 0.7 (0.6 - 0.8) | 0.364 |
|  | Dominant hand Mean velociy [mm/s] | 28.2 (23.7 - 32.3) | 27.6 (22.9 - 33.8) | 0.772 |
|  | Dominant hand Mean acceleration [mm/s^2^] | 4 (1.8 - 7) | 3.7 (2 - 7) | 0.908 |
|  | Non-dominant hand Path length [cm] | 4787.3 (3819.1 - 6136.9) | 5123.3 (3483.6 - 6687.7) | 0.862 |
|  | Non-dominant hand Idle time [%] | 0.7 (0.6 - 0.8) | 0.7 (0.7 - 0.8) | 0.511 |
|  | Non-dominant hand Mean velociy [mm/s] | 24.3 (20.8 - 29.6) | 27.2 (23.4 - 29.8) | 0.334 |
|  | Non-dominant hand Mean acceleration [mm/^2^] | 3.4 (2.5 - 5.5) | 3 (1.6 - 4.7) | 0.147 |
| **Laparoscopic Suture and Knot** | |  |  |  |
|  | Task time [sec] | 212.7 (148.2 - 273.7) | 252.1 (171.4 - 320.9) | 0.195 |
|  | Motion volume both instruments [cm³] | 1501 (1117 - 2459) | 1763 (1456 - 2434) | 0.417 |
|  | Relative time instruments out of view [%] | 14 (3 - 24) | 2 (1 - 5) | **0.002** |
|  | Dominant hand Path length [cm] | 4151.8 (2453.2 - 6115) | 5572.8 (3418.3 - 7489) | 0.086 |
|  | Dominant hand Idle time [%] | 0.7 (0.6 - 0.7) | 0.7 (0.6 - 0.7) | 0.463 |
|  | Dominant hand Mean velociy [mm/s] | 24.8 (20.6 - 28.5) | 25.4 (23.6 - 30.9) | 0.254 |
|  | Dominant hand Mean acceleration [mm/s^2^] | 5.3 (3.1 - 9.6) | 5.2 (3.7 - 8.6) | 0.938 |
|  | Non-dominant hand Path length [cm] | 2002.7 (1390.1 - 5120.9) | 4186.9 (2180.4 - 6667.1) | **0.042** |
|  | Non-dominant hand Idle time [%] | 0.6 (0.5 - 0.7) | 0.6 (0.6 - 0.7) | 0.575 |
|  | Non-dominant hand Mean velociy [mm/s] | 32.9 (25.2 - 35.4) | 31.2 (27 - 34.9) | 0.832 |
|  | Non-dominant hand Mean acceleration [mm/^2^] | 15 (9.9 - 32.6) | 11.6 (7.6 - 15) | 0.075 |

Supplementary Table 3: Comparison of test results (Test 3) between participants with previous laparoscopic experience vs. participants with no laparoscopic experience (significant p-values markes bold)

|  |  | **Test 4** | | |
| --- | --- | --- | --- | --- |
|  |  | **previous laparoscopic  experience** | **no laparoscopic  experience** | **p-value** |
|  |  | Median (IQR) | Median (IQR) |  |
| **Peg-Transfer** | |  |  |  |
|  | Task time [sec] | 119.7 (104.3 - 124.5) | 127.3 (109.7 - 150.1) | 0.068 |
|  | Motion volume both instruments [cm³] | 1122 (963 - 1345) | 1304 (962 - 1734) | 0.162 |
|  | Relative time instruments out of view [%] | 1 (0 - 1) | 2 (0 - 5) | 0.121 |
|  | Dominant hand Path length [cm] | 3908.6 (3516.9 - 4263.4) | 4257.2 (3695.3 - 5302.7) | 0.098 |
|  | Dominant hand Idle time [%] | 0.6 (0.5 - 0.6) | 0.6 (0.5 - 0.6) | 0.379 |
|  | Dominant hand Mean velociy [mm/s] | 34.5 (31.3 - 37.6) | 35 (31.4 - 37.6) | 0.488 |
|  | Dominant hand Mean acceleration [mm/s^2^] | 1.6 (0.6 - 2.8) | 1.4 (0.7 - 2.5) | 0.704 |
|  | Non-dominant hand Path length [cm] | 4103 (3488.1 - 4878.2) | 4570.8 (3774 - 5413.3) | 0.271 |
|  | Non-dominant hand Idle time [%] | 0.5 (0.4 - 0.6) | 0.5 (0.4 - 0.6) | 0.846 |
|  | Non-dominant hand Mean velociy [mm/s] | 35.2 (33.1 - 42.5) | 37.8 (30.9 - 44.1) | 0.832 |
|  | Non-dominant hand Mean acceleration [mm/^2^] | 2.4 (1 - 5.7) | 3.2 (1.5 - 6.1) | 0.89 |
| **Precision Cut** | |  |  |  |
|  | Task time [sec] | 161.9 (115.6 - 179) | 184.2 (139.8 - 200.6) | 0.122 |
|  | Motion volume both instruments [cm³] | 1406 (849 - 1612) | 1270 (950 - 1907) | 0.623 |
|  | Relative time instruments out of view [%] | 5 (1 - 24) | 4 (0.5 - 13) | 0.595 |
|  | Dominant hand Path length [cm] | 4116.5 (2091.5 - 5337.5) | 4805.5 (2915.3 - 6885.9) | 0.122 |
|  | Dominant hand Idle time [%] | 0.5 (0.4 - 0.7) | 0.6 (0.5 - 0.8) | 0.476 |
|  | Dominant hand Mean velociy [mm/s] | 30.1 (20.2 - 39) | 31.1 (20.1 - 39.7) | 0.934 |
|  | Dominant hand Mean acceleration [mm/s^2^] | 4.4 (2.3 - 8.5) | 2.7 (1.2 - 4.2) | **0.025** |
|  | Non-dominant hand Path length [cm] | 5202 (4004.4 - 6399.4) | 5622.6 (4316 - 7206.3) | 0.443 |
|  | Non-dominant hand Idle time [%] | 0.6 (0.5 - 0.7) | 0.5 (0.5 - 0.7) | 0.821 |
|  | Non-dominant hand Mean velociy [mm/s] | 32.2 (30.6 - 40.9) | 32.3 (27.9 - 40.8) | 0.718 |
|  | Non-dominant hand Mean acceleration [mm/^2^] | 3.7 (1.7 - 7.3) | 2.7 (1.3 - 4.1) | 0.219 |
| **Ballon Resection** | |  |  |  |
|  | Task time [sec] | 172.5 (155.7 - 287.3) | 181.3 (153.5 - 262.8) | 0.774 |
|  | Motion volume both instruments [cm³] | 1719 (1498 - 2294) | 1746 (1334 - 2396) | 0.934 |
|  | Relative time instruments out of view [%] | 4 (2 - 9) | 6 (1.5 - 12.5) | 0.452 |
|  | Dominant hand Path length [cm] | 4230.6 (2907.6 - 7504.6) | 4596.3 (3395.4 - 6558.8) | 0.624 |
|  | Dominant hand Idle time [%] | 0.7 (0.6 - 0.7) | 0.7 (0.6 - 0.7) | 0.788 |
|  | Dominant hand Mean velociy [mm/s] | 28.1 (26.3 - 29.9) | 26.2 (22.5 - 32.5) | 0.421 |
|  | Dominant hand Mean acceleration [mm/s^2^] | 3.2 (1.4 - 7) | 3.9 (2.3 - 5.7) | 0.993 |
|  | Non-dominant hand Path length [cm] | 3841.4 (3551.8 - 6287.3) | 4588.6 (3539.4 - 7010.1) | 0.465 |
|  | Non-dominant hand Idle time [%] | 0.7 (0.6 - 0.8) | 0.7 (0.6 - 0.8) | 0.465 |
|  | Non-dominant hand Mean velociy [mm/s] | 24.4 (21.9 - 30.7) | 26.5 (22.4 - 33.8) | 0.465 |
|  | Non-dominant hand Mean acceleration [mm/^2^] | 2 (0.7 - 4.7) | 2.8 (1.9 - 5.1) | 0.263 |
| **Laparoscopic Suture and Knot** | |  |  |  |
|  | Task time [sec] | 243.1 (158.9 - 316.8) | 207.5 (172.7 - 282.4) | 0.803 |
|  | Motion volume both instruments [cm³] | 2297 (1831 - 2454) | 2195 (1432 - 2588) | 0.611 |
|  | Relative time instruments out of view [%] | 3 (2 - 7) | 3 (1.5 - 6.5) | 0.533 |
|  | Dominant hand Path length [cm] | 4602.3 (3798.3 - 6371.4) | 4335 (3342.2 - 7241.6) | 0.86 |
|  | Dominant hand Idle time [%] | 0.7 (0.6 - 0.7) | 0.7 (0.6 - 0.7) | 0.41 |
|  | Dominant hand Mean velociy [mm/s] | 28.4 (22.8 - 32.8) | 27.4 (24.5 - 32.6) | 0.774 |
|  | Dominant hand Mean acceleration [mm/s^2^] | 5.7 (2.9 - 10.2) | 5.9 (4.4 - 8.4) | 0.76 |
|  | Non-dominant hand Path length [cm] | 3266.7 (2354.6 - 5159.8) | 3047.4 (2383.9 - 5315.8) | 0.86 |
|  | Non-dominant hand Idle time [%] | 0.6 (0.6 - 0.6) | 0.6 (0.6 - 0.7) | 0.86 |
|  | Non-dominant hand Mean velociy [mm/s] | 32.2 (28.9 - 36.2) | 32.6 (28.4 - 36.7) | 0.963 |
|  | Non-dominant hand Mean acceleration [mm/^2^] | 12.1 (10.6 - 17.9) | 13.5 (9.3 - 19.9) | 0.919 |

Supplementary Table 4: Comparison of test results (Test 4) between participants with previous laparoscopic experience vs. participants with no laparoscopic experience (significant p-values markes bold)
